# Molecular epidemiology of *Bordetella pertussis* and analysis of vaccine antigen genes from clinical isolates from Shenzhen, China

**DOI:** 10.1186/s12941-021-00458-3

**Published:** 2021-08-18

**Authors:** Shuang Wu, Qinghua Hu, Chao Yang, Haijian Zhou, Hongyu Chen, Yanwei Zhang, Min Jiang, Yuxiang He, Xiaolu Shi

**Affiliations:** 1 Shenzhen Center for Disease Control and Prevention, 8 Longyuan Road, Nanshan District, Shenzhen, China; 2 National Institute for Communicable Disease Control and Prevention (ICDC) of China CDC, Beijing, China; 3 Shenzhen Children’s Hospital, Shenzhen, China

**Keywords:** *Bordetella pertussis*, Genotype, Pertussis vaccine, Molecular epidemiology, Erythromycin-resistant

## Abstract

**Background:**

Although pertussis cases globally have been controlled through the Expanded Programme on Immunization (EPI), the incidence of pertussis has increased significantly in recent years, with a “resurgence” of pertussis occurring in developed countries with high immunization coverage. Attracted by its fast-developing economy, the population of Shenzhen has reached 14 million and has become one of the top five largest cities by population size in China. The incidence of pertussis here was about 2.02/100,000, far exceeding that of the whole province and the whole country (both < 1/100,000). There are increasing numbers of reports demonstrating variation in *Bordetella pertussis* antigens and genes, which may be associated with the increased incidence. Fifty strains of *Bordetella pertussis* isolated from 387 suspected cases were collected in Shenzhen in 2018 for genotypic and molecular epidemiological analysis.

**Methods:**

There were 387 suspected cases of pertussis enrolled at surveillance sites in Shenzhen from June to August 2018. Nasopharyngeal swabs from suspected pertussis cases were collected for bacterial culture and the identity of putative *Bordetella pertussis* isolates was confirmed by real-time PCR. The immunization history of each patient was taken. The acellular pertussis vaccine (APV) antigen genes for pertussis toxin (*ptxA, ptxC*), pertactin (*prn*) and fimbriae (*fim2* and *fim3)* together with the pertussis toxin promoter region (*ptxP*) were analyzed by second-generation sequencing. Genetic and phylogenetic analysis was performed using sequences publicly available from GenBank, National Institutes of Health, Bethesda, MD, USA (https://www.ncbi.nlm.nih.gov/genbank/). The antimicrobial susceptibility was test by Kirby-Bauer disk diffusion.

**Results:**

Fifty strains of *Bordetella pertussis* were successfully isolated from nasopharyngeal swabs of 387 suspected cases, with a positivity rate of 16.79%, including 28 males and 22 females, accounting for 56.0% and 44.0% respectively. Thirty-eight of the 50 (76%) patients were found to be positive for B. pertussis by culture. Among the positive cases with a history of vaccination, 30 of 42 (71.4%) cases had an incomplete pertussis vaccination history according to the national recommendation. Three phylogenetic groups (PG1-PG3) were identified each containing a predominant genotype. The two vaccines strains, CS and Tohama I, were distantly related to these three groups. Thirty-one out of fifty (62%) isolates belonged to genotype PG1, with the allelic profile *prn2/ptxC2/ptxP3/ptxA1/fim3-1/fim2-1*. Eighteen out of fifty (36%) isolates contained the A2047G mutation and were highly resistant to erythromycin, and all belonged to genotype PG3 (*prn1/ptxA1/ptxP1/ptxC1/fim3-1/fim2-1*), which is closely related to the recent epidemic strains found in northern China.

**Conclusions:**

The positive rate of cases under one-year-old was significantly higher than that of other age groups and should be monitored. The dominant antigen genotypes of 50 Shenzhen isolates are closely related to the epidemic strains in the United States, Australia and many countries in Europe. Despite high rates of immunization with APV, epidemics of pertussis have recently occurred in these countries. Therefore, genomic analysis of circulating isolates of *B. pertussis* should be continued, for it will benefit the control of whooping cough and development of improved vaccines and therapeutic strategies.

## Background

Pertussis is an acute respiratory infectious disease caused by the gram-negative bacterium *Bordetella pertussis* [1]. The typical symptoms of pertussis include paroxysmal coughing with an inspiratory whoop, post-tussive vomiting, cyanosis, and persistent coryzal symptoms [2]. Pertussis is mainly transmitted by droplets and older children and adults have been increasingly recognized as reservoirs of infection for transmission to unvaccinated or incompletely vaccinated infants who are most at risk of severe disease complications and death [3]. The first pertussis whole-cell vaccine (WPV) made from killed cells was licensed in the USA in 1914. Later chemical inactivation was used and the WPV combined with tetanus and diphtheria toxoids in the 1940s to become widely used diphtheria-tetanus-pertussis (DTP) vaccine, which is about 80% effective in preventing severe illness and death from pertussis. Since then, a successive decline in the incidence of the disease has been observed [4]. However, due to the reactogenicity following WPV immunization (including fever, limb redness and swelling) and the incorrect public opinion linking the WPV with encephalopathies, many parents refused to vaccinate their children, vaccine coverage fell and lawsuits against the vaccine manufacturers forced many of them to stop producing the vaccine [5, 6]. To address the reactogenicity of WPV, vaccine manufacturers switched to producing APV containing a number of highly purified antigens [1]. Since then, there has been an increase in whooping cough cases and a dramatic cycle of epidemics [7, 8]. According to the World Health Organization (WHO), as many as 195,000 children worldwide died of pertussis and its complications in 2008, with 90% of cases occurring in less developed and developing countries [9]. Even in developed countries or countries with high pertussis vaccination rates, the incidence of pertussis has been on the rise in recent years [10]. In 2012, there was an outbreak of pertussis in Washington and other states in the United States, with the highest reported incidence (37.5/100,000) since 1942. It is worth noting that 43% of patients had at least four doses of immunological history of acellular diphtheria, tetanus and pertussis (DTaP) vaccine [11]. According to the different production methods, the common DTaP vaccine used in China can be divided into two types: co-purified vaccines and component vaccines. Domestic manufacturers mainly produced co-purified vaccines. Since there was no separate purification of different antigen components during production procedure, the proportion of antigens in the original solution varied according to the manufacturers and production batches. A study has pointed out that the co-purified pertussis vaccines produced in China contained not only the main antigens purified pertussis toxoid (PT), filamentous hemagglutinin (FHA) and pertactin (PRN), but also fimbriae (FIM) 2 and 3 and other minor protein antigens [12]. For component vaccines, PT, FHA, PRN and other antigens were extracted separately by column chromatography, and then were mixed in a certain proportion, which ensured the consistent quality between batches and had higher purity. Only some imported DTaP vaccines (Pentaxim, Sanofi Pasteur, France and Infanrix, GlaxoSmithKline, Britain) used this technology for production. However, the use of imported vaccines were limited by their high price [13]. Historically, the intracerebral mouse protection test (Kendrick test) has been used to effective determine the potency of whole-cell pertussis vaccines and is the only test that has shown a correlation with protection in children [14].

In recent years, the reported incidence of pertussis in adolescents and adult has increased, due to a combination of better awareness of less severe clinical symptoms and increased availability of diagnostic testing including PCR and serology [15, 16]. Vaccines are the main way to prevent pertussis, which have been widely used for immunization in China since 1960s [17]. Shenzhen adopted whole-cell vaccines before 2008, and gradually replaced it with APV from 2008 to 2010. Since 2010, APV have been used throughout the city. According to the national immunization program, the the recommended age for receipt of the DTaP is 3, 4, 5 and 18 months after birth [18]. The vaccination rate has kept above 99% in recent years. However, according to the statistics of the China Information System for Disease Control and Prevention, the number of reported cases of pertussis in Shenzhen is rising sharply. Studies have shown that pathogens have undergone adaptive changes under the pressure of immune protection pressure induced by the body after vaccination. The alleles of the genes encoding the vaccine antigens including PT, PRN, FIM in the predominant circulating strains in Shenzhen are distinct from the vaccine strains [19, 20].

To further explain the possible impact of the genetic difference on the immune effects, and to better understand the pathogenic characteristics, evolutionary characteristics, and molecular epidemiology of *Bordetella pertussis* in China, we sequenced 50 strains of *Bordetella pertussis* isolated from Shenzhen in 2018 to analyze their population structure and sequence characteristics of the major vaccine antigen genes.

## Materials and methods

### Research object

Three hundred and eight-seven suspected pertussis cases from the outpatient department of Shenzhen Children’s Hospital from June to August 2018.

### Main reagents and instruments

#### Main reagents

Charcoal Agar Base and Bordetella selective supplement (Oxoid company, Canada), Pertussis Bacteria Phase I Standard Serum (REMEL company, Lenexa City, KS, USA) and Whole-genome DNA extraction kit (Qiagen Company, Shanghai, China).

#### Main instruments

Fluorescence PCR instrument (ABI Company, Oyster Bay, NY, USA) and NanoDrop 1000 Ultra Micro Spectrophotometer (Thermo Fisher Company, Fair Lawn, NJ, USA).

#### Culture

Charcoal agar was prepared according to the instructions of the manufacturer. After autoclaving and cooling to 50 °C, Bordetella selective supplement and 10% defibrinated sheep blood were added into the agar. The samples were inoculated onto the charcoal blood agar plates and incubated at 37 °C for 3 days. Based on colonial appearance, round, moist, protruding, transparent or translucent, presumptive *Bordetella pertussis* colonies were subcultured to obtain pure cultures and identified by biochemical reactions and agglutination tests. Genomic DNA was extracted from the presumptive *B. pertusiss* isolates and tested by real-time PCR using the insertion element IS*481* and pertussis toxin subunit A gene targets [21].

#### Genome sequencing and de novo assembly

The genomic DNA of *Bordetella pertussis* was extracted using the whole-genome DNA extraction kits according to the manufacturer’s instructions. The library was constructed using the Illumina HiSeq 4000 system (Illumina, San Diego, CA, USA) at the Beijing Genomics Institute (Shenzhen, China). DNA sample preparation kit (Illumina, San Diego, CA, USA), and whole gene sequencing and preliminary evaluation filtering was conducted using the Illuminas HiSeq X-Ten platform (BGI, Shenzhen, China). The original data was further filtered by SOAPnuke to obtain valid data via quality control. De novo assembly of the genome was performed by SPAdes gene assembly software (V3.9.1).

#### Sequence analysis of major antigen genes

The sequence numbers of the reference genes are as follows:

*ptxA* gene (vaccine CS strain [22] *ptxA2* type: WP_010931648.1, Tohama I strain *ptxA2* type: NP_882282.1, B592 strain *ptxA2* type: AJ245367.1, 287 strain *ptxA*1 type: AJ006155.1, B6 strain *ptxA*4 type: AJ506336.1);

*ptxC* gene (Tohama I strain *ptxC1* type:NP_882286.1, 3779 strain *ptxC1* type: AAA22985.1, NK strain *ptx*C2 type: AJ420987.1);

*ptxP* gene (Tohama I strain *ptx*P1 type: FN252323.1, B2983 strain *ptx*P3 type: FN252324.1);

*prn* gene(vaccine CS strain:WP_010930159 0.1, Tohama I strain: NP_879839.1, B391 strain *prn1* type: AJ011091.1, B345 strain *prn2* type: AJ011092.1, B343 strain *prn3* type: AJ011093.1);

*fim2* gene (vaccine CS strain *fim2-1* type: WP_010930199.1, Tohama I strain: NP_879898.1, NK strain *fim*2-2 type: AJ420988.1);

*fim3* gene (vaccine CS strain: WP_010930436.1, Tohama I strain *fim3-1* type: NP_880302.1, *fim3-2* type: AY464179.1, *fim3-3* type:AY464180.1, *fim3-4* type: AY464181.1). The homologous sequences of virulence factors in the genome sequence of Shenzhen strains were extracted using Blastn, and the sequence alignment and phylogenetic analysis of the homologous genes of *ptxA, ptxC, ptxP, prn, fim2* and *fim3* sequences was performed using Mega 7.0 software. Genomic relationships of the major antigen was visualize by using GrapeTree software [23].

### Antimicrobial susceptibility tests

We thawed fifty B. pertussis isolates on the charcoal agar which containing 10% sheep blood for 4 days. Then subcultured the bacterial suspension with 0.5 McFarland standard on the same kind of culture medium for 72 h. Kirby-Bauer disk diffusion test was used to determine susceptibility to erythromycin. An inhibition diameter > 42 mm suggested complete susceptibility to erythromycin according to some studies [24]. *Staphylococcus aureus* ATCC25923 and *B. pertussis* ATCC9797 were used as controls.

### Single nucleotide polymorphism (SNP) extraction and phylogenetic typing

Eight hundred and forty-two public genome sequences were downloaded from GenBank (https://www.ncbi.nlm.nih.gov/genome/browse#!/prokaryotes/1008/, as of September 2020). Snippy software (https://github.com/tseemann/snippy/) was used to detect the core genome SNP sites of the public sequences and the Shenzhen strains sequences, and the genome sequence of Tohama I strain (GenBank Number: BX470248.1) was used as a reference. TRF software (https://tandem.bu.edu/trf/trf.html) and BLASTn were used to identify the recombination region of the reference genome and to remove the SNP sites located in the repeating region. Based on non-repetitive regions of SNP sites, the maximum likelihood phylogenetic tree was built using IQ-Tree software.

## Results

### Laboratory test results and epidemiological characteristics analysis

Fifty strains of *Bordetella pertussis* were obtained from nasopharyngeal swabs of 387 children with bedside inoculation, with a positivity rate of 16.79% (65/387). The epidemiological characteristics of patients are shown in Table 1. The vaccination history of eight out of 50 cases was not available. Of the remaining 42 cases, 11 (26.2%) had not received any pertussis-containing vaccine, 5 (11.9%) had completed the primary course (three doses) and 7 (16.7%) had also received a booster (Table 1). Altogether, 30 of 42 (71.4%) cases have an incomplete pertussis vaccination history according to the national recommendation, 14 of 30 (46.7%) cases have not received the recommended number of immunizations according to their age, while the other 16 cases were infected before the recommended age for vaccination.

**Table 1:** Epidemiological characteristics and vaccination status of children

Number of immunizations	Number of cases	Gender		Age (months)			
		Male	female	0–3	4–6	7–12	> 12
0	11	8	3	9	2	0	0
1	13	6	7	1	9	2	1
2	6	3	3	1	2	2	1
3	5	2	3	0	0	2	3
4	7	3	4	0	0	0	7
Unknown	8	6	2	1	2	3	1

### Genotyping and erythromycin-resistant of *B. pertussis* isolates

**Fig. 1:**
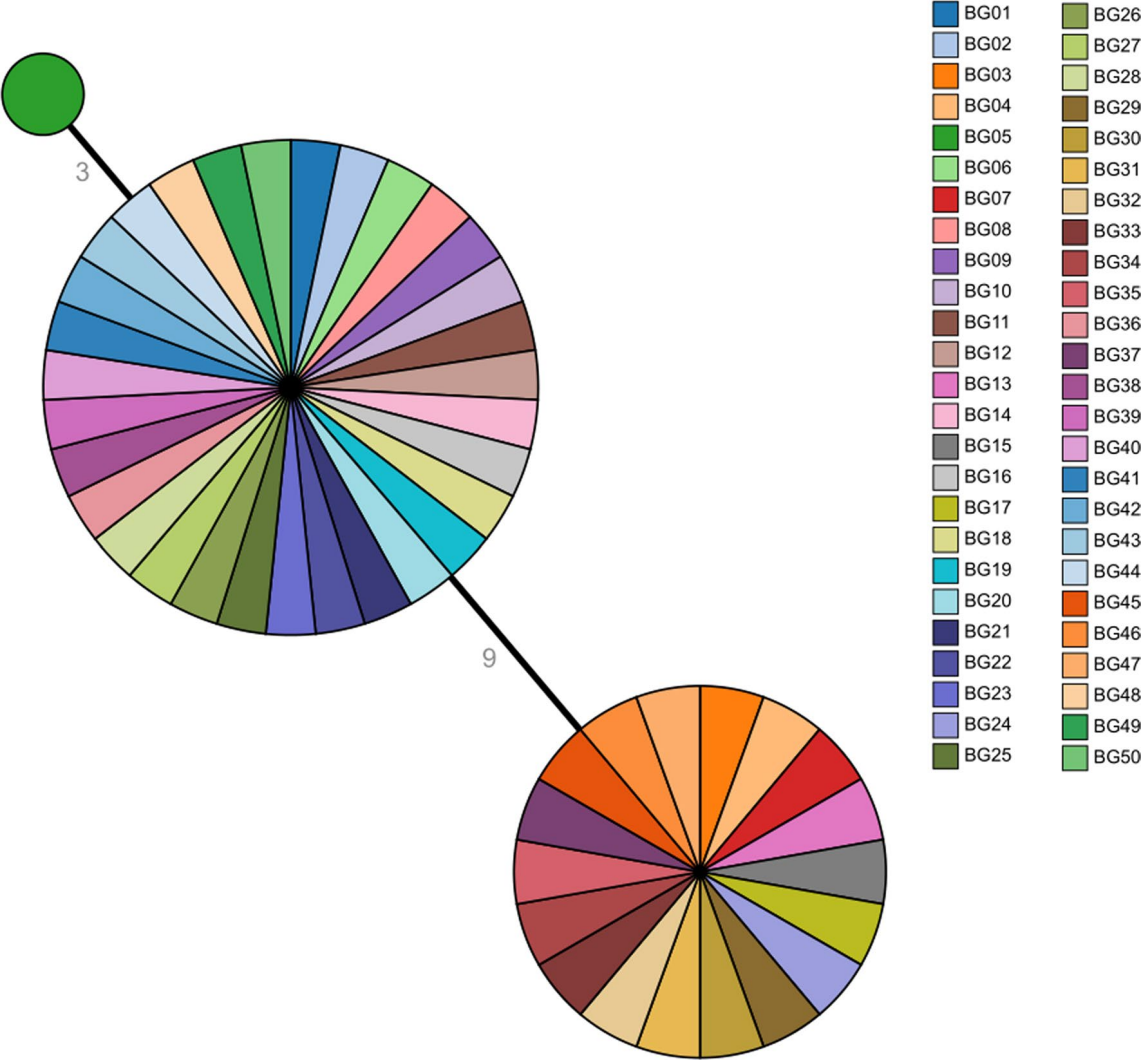
The minimum spanning tree of 50 Shenzhen stains. The size of the circle indicates the number of strains with this particular genotype. Each color represents one sample. The length of the connecting lines between the circles indicates the genetic distance between different genotypes

Analysis of the six genes revealed that two had 100% similarity (1242/1242 bases) with those in the vaccine strains CS and Tohama l, namely *fim2-1* and *fim3-1* respectively. The nucleotide sequences of the remaining four genes were distinct and non-synonymous resulting in a change in antigen type. All *ptxA* gene sequences in the Shenzen isolates had non-synonymous mutations. All the clinical isolates had the alleleic profile *ptxA1* and were distinct from the vaccine strain allele, *ptxA2*. Non-synonymous mutations also occurred in the pertussis toxin promoter gene (*ptxP*). Nineteen strains had the same allelic sequence as the vaccine strain (*ptxP1*), and the remaining 31 were *ptxP3*. Synonymous mutations were found in the pertussis toxin S3 subunit gene (*ptxC*). Thirty-one isolates had the allelic profile *ptxC2* (Table 2). Three allelic profiles of the pertactin gene (*prn*) were found in the 50 isolates including, *prn1* (n = 18), *prn2* (n = 31) and *prn3* (n = 1). The major genotypes of the 50 isolates can be divided into three categories, *prn1/ptxC1/ptxP1/ptxA1/ fim3-1/fim2-1* (18/50, 36%), *prn2/ptxC2/ptxP3/ptxA1/fim3-1/fim2-1*(31/50, 62%) and *prn3/ptxC1/ ptxP1/ptxA1/fim3-1/fim2-1* (1/50, 2%) (Table 2). Each circle of the minimum spanning tree represents an genotype of the major antigen and discern the genetic relationship among three categories (Fig. 1). All the 18 strains with A2047G mutation site (PG3) were erythromycin resistant while the other 32 strains were sensitive to erythromycin (zone of inhibition > 42 mm).

**Table 2:** Main virulence genotypes of clinical isolates in Shenzhen

Genotype	Strain number	Proportion
*prn1/ptxA1/ptxP1/ptxC1/fim3-1/fim2-1*	BG03,04,07,13,15,17,24,29,30,31,32,33,34,35,37, 45,46,47	18 (36%)
*prn2/ptxA1/ptxP3/ptxC2/fim3-1/fim2-1*	BG01,02,06,08,09,10,11,12,14,16,18,19,20,21,22, 23,25,26,27,28,36,38,39,40,41,42,43,44,48,49,50	31 (62%)
*prn3/ptxA1/ptxP1/ptxC1/fim3-1/fim2-1*	BG05	1 (2%)
*prn1/ptxA2/ptxP1/ptxC1/fim3-1/fim2-1*	CS^a^, TohamaI^b^	0 (0%)

^a^CS strain is a Chinese strain isolated in Beijing and used for production of pertussis vaccine

^b^Tohama I strain is a strain isolated in Japan in the 1950s and widely used for production of pertussis vaccine.c These genes encode: pertactin (*prn*); pertussis toxin subunits S1 (*ptxA*) and S3 *(ptxC*); pertussis toxin promoter region (*ptxP*); fimbrial antigen 2 (*fim2*) and 3 (*fim3*)

### Genome evolution analysis

**Fig. 2:**
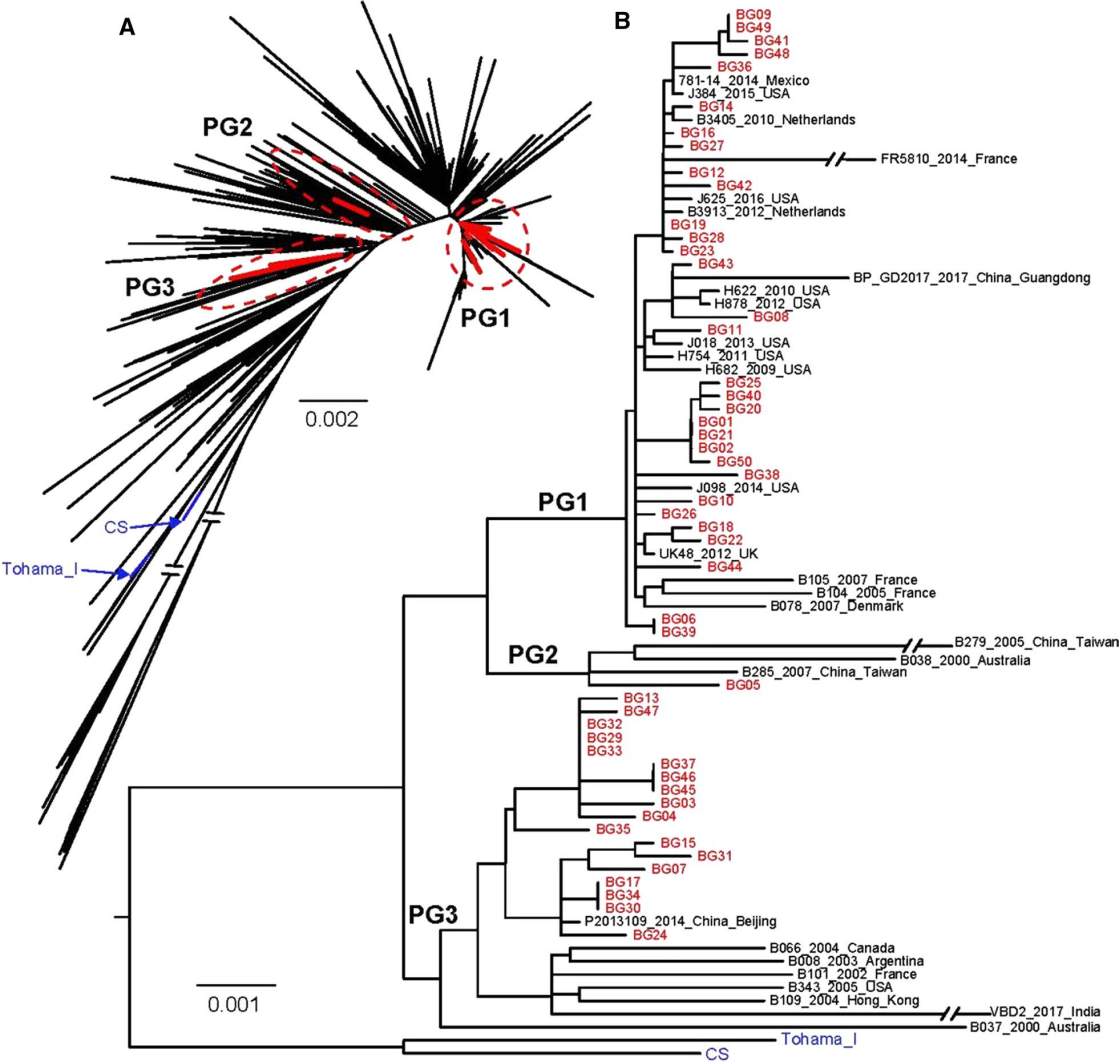
The phylogenetic tree of Shenzhen strains and international strains. **A** Maximum likelihood phylogenetic tree of 50 Shenzhen strains and 842 international strains. **B** Maximum likelihood phylogeny tree of Shenzhen strain, phylogenetic branch PG1-PG3 strain and reference strain. Red and blue represented the Shenzhen strain and two reference strains, respectively. For better visualization, the longer phylogenetic branches were artificially shortened (double slashes)

Genetic analysis was performed on the whole genome sequences from the 50 study isolates, the CS and Tohama I strain and 842 publicly available *B. pertussis* strains (Fig. 2). The Shenzhen isolates in this study were distributed in three evolutionary branches (phylogenetic group, PG1 to 3). The genotype *prn2/ptxA1/ptxP3/ptxC2/ fim3-1 /fim2-1* predominated in PG1, genotype *prn3/ptxA1/ptxP1/ptxC1/fim3-1/fim2-1* in PG2, and genotype *prn1/ptxA1/ptxP1/ptxC1/fim3-1/fim2-1* in PG3. The isolates in PG3 were closely related to the epidemic strains in northern China in recent years, but distant from foreign strains. All isolates were genetically distant from vaccine strains CS and Tohama I (Fig. 2).

## Discussion

*Bordetella pertussis* is the only species in the genus *Bordetella* that can produce PT, which is its major virulence factor and consists of five subunits (S1 to S5). Pertussis toxin belongs to a class of toxins known as A-B toxins. The A domain contains the S1 subunit which has immunoprotective properties and a variety of enzyme activities, and is expressed by *ptxA* gene [21]. The region between amino acid 65 and 233 is the conserved sequence of pertussis toxin, causing the host cell immune response and is the antigen recognition site of T cells. According to the nucleotide differences of the 68th, 228th, and 232nd amino acids of S1, the ptxA gene is divided into four allelic types, 1, 2, 3 and 4 [22]. The *ptxA* gene subtypes of pertussis vaccine strains and strains isolated before or early in vaccination were allelic types *ptxA2* or *ptxA4* in many countries. The reference strains Tohama I and CS in this study belong to the *ptxA2* allele, while all of the 50 isolates from Shenzhen had non-synonymous mutations (A to G), that is, from the original isoleucine (I), coded for by ATA to methionine (M), coded for by ATG and belong to the *ptxA1* allele.The protective effect of the vaccine produced by the vaccine containing the *ptxA*2 vaccine strain is weak against the strain containing the *ptxA*1 gene [25]. The pertussis toxin promoter (*ptxP*) region is close to 170 bases located upstream of the pertussis toxin gene in the *B. pertussis* genome. The lack of expression of pertussis toxin in *B. parapertussis* and *B. bronchiseptica* is due to differences in the promoter regions of the operons, which have species-specific differences [26]. This region contains binding sites for RNA polymerase and 6 binding sites of BvgA regulatory protein dimers, which can promote the transcriptional expression of PT by interacting with the regulatory protein BvgA. In recent years, researchers have discovered that *Bordetella pertussis ptxP* presents a certain polymorphism, and according to the mutation bases at specific positions, *Bordetella pertussis ptxP* is divided into 11 subtypes. Dutch researchers have confirmed that strains containing *ptxP*3 can highly express PT and have gradually become the dominant epidemic strains in the Netherlands. It is speculated that the “pertussis recurrence” may be related to this. Therefore, the *ptxP* gene of *Bordetella pertussis* has attracted more and more attention and has been used as a molecular genetic marker for the evolution of *Bordetella pertussis* [10]. The *ptxP3* allele accounted for 62% of the 50 pertussis strains isolated in this study, which is consistent with the international trend.

The pertactin protein is a non-ciliary outer membrane protein produced by *Bordetella pertussis*, as an important virulence factor, PRN protein plays an important role in bacterial infection and adhesion to the epithelial cell membrane of the host respiratory system. PRN protein is also an important protective antigen that can induce humoral and cellular immune responses in mice. Pertactin has shown a good protection rate in the respiratory tract attack animal model test of *Bordetella pertussis* [21]. According to the difference in the structure of this region and amino acids at specific positions, the *prn* gene is divided into eighteen allelic types *prn1-18* [22, 27, 28]. Studies in some countries have confirmed that *prn* genotypes of strains isolated before or in the early stages of inoculation and pertussis vaccine strain are mainly *prn1* genotypes. Since the 1980s, *prn2* subtypes have appeared in some European countries. By 2000s, *prn2 or prn3* had been the dominant genotypes of the isolates in the Netherlands, Finland, France, the United States, and other countries [29, 30]. Bioinformatics analyses of the amino acid secondary structure deduced from the *prn* gene sequence of strains isolated in different ages show that the *prn* genotype structure of the strains isolated after the 2000s changed to a certain extent, and the hydrophilic region appeared, which led to the change of its immunogenicity. However, the previously isolated *prn1*, *prn7*, *prn10* and *prn11* are completely hydrophobic region in this region 1 [31]. In this study, 62% of the isolates belonged to *prn2* and 2% belonged to *prn3*, which was consistent with the evolution trend of *prn* genes in the world.

Clinical trials in some countries have reported that there is a good correlation between the anti-Fim protein antibody titer in the pertussis vaccine and the immune response in mice and the immune vaccine protection in the children. WHO guidelines for pertussis vaccine recommend that strains expressing of type 2 and 3 Fim protein antigens should be selected as the production strain in the pertussis vaccine production. As reported, these two fimbriae proteins also have certain immunogenicity and have been used as components of APV by some vaccine manufacturers. In this study, the isolates contained the same *fim2-1* and *fim3-1* genes as the vaccine strain, indicating that these two antigen-related genes are relatively conservative.

In this study, only 16.7% of the children had completed the full vaccination of pertussis vaccine, and about 71.4% of positive patients had not completed the basic process of APV vaccination at the time of onset, mainly because these children were relatively young at the age of onset and had not yet reached the planned immunization. The current pertussis immunization targets in China do not include pregnant women, resulting in infants receiving little pertussis antibody from the mother, and the first dose of vaccination for the child is 3 months old, leading to a longer unprotected window a long-unprotected window for infants of younger months. These may be important reasons for the high incidence of pertussis in China. Another possible reason is that the effectiveness of the pertussis vaccine declines rapidly over time [32, 33]. Studies have shown that the pertussis vaccine is highly effective within three years of vaccination, but then the immunity gradually weakens, with little protection after 7 years [34, 35].

Earlier studies have shown that the genotypes of the pertussis strains were different from those of the vaccine strains, and the hypothesis of pertussis antigenic shifts was put forward. It was hypothesized that changes in the bacterial genes were driven by immune selection pressure leading to the increase of these main epidemic strains. At the same time, the researchers also speculated that due to the antigenic drift of *B. pertussis*, the antigenic protein subtypes between the vaccine strains and clinical isolates were different, which weaken the immunity effect of pertussis vaccine, and may also be one of the reasons for the reemergence of pertussis in highly vaccinated countries [25]. The genetic diversity of *B. pertussis* varied greatly prior to mass vaccination with WCVs, and significantly decreased from the 1990s, followed by clonal expansion coinciding with the epidemic periods [36]. Therefore, molecular monitoring of epidemic strains is particularly important. This study recommends that whole genome sequencing should be used to add information on pertussis epidemic strains in different countries or regions to the established database, and to gradually establish a pertussis epidemic surveillance system that can be shared by global public health departments and researchers.

## Conclusions

In conclusion, this study presents some explanations for pertussis resurgence, including improved laboratory detection methods and adaptation of *Bordetella pertussis*. The dominant antigen genotype is *prn2/ptxC2/ptxP3/ptxA1/ fim3-1/fim2-1*, which is closely related to the epidemic strains in the United States, Australia and many countries in Europe. Despite high rates of immunization with APV, epidemics of pertussis have recently occurred in these countries. Therefore, genomic analysis of circulating isolates of *B. pertussis* should be continued, for it will benefit the control of whooping cough and development of improved vaccines and therapeutic strategies.

## Data Availability

The data that support the findings of this study have been deposited into CNGB Sequence Archive (CNSA) [34] of China National GeneBank DataBase (CNGBdb) [35] with Accession Number CNP0001528. The datasets generated for this study are available on request to the corresponding author.
